# Temporal order judgment of multisensory stimuli in rat and human

**DOI:** 10.3389/fnbeh.2022.1070452

**Published:** 2023-01-12

**Authors:** Fatemeh Mafi, Matthew F. Tang, Mohammad Reza Afarinesh, Sadegh Ghasemian, Vahid Sheibani, Ehsan Arabzadeh

**Affiliations:** ^1^Neuroscience Research Center, Institute of Neuropharmacology, Kerman University of Medical Sciences, Kerman, Iran; ^2^Cognitive Neuroscience Research Center, Institute of Neuropharmacology, Kerman University of Medical Sciences, Kerman, Iran; ^3^Eccles Institute of Neuroscience, John Curtin School of Medical Research, The Australian National University, Canberra, ACT, Australia

**Keywords:** audiovisual, temporal order judgment, temporal precision, temporal sensitivity, speed-accuracy

## Abstract

We do not fully understand the resolution at which temporal information is processed by different species. Here we employed a temporal order judgment (TOJ) task in rats and humans to test the temporal precision with which these species can detect the order of presentation of simple stimuli across two modalities of vision and audition. Both species reported the order of audiovisual stimuli when they were presented from a central location at a range of stimulus onset asynchronies (SOA)s. While both species could reliably distinguish the temporal order of stimuli based on their sensory content (i.e., the modality label), rats outperformed humans at short SOAs (less than 100 ms) whereas humans outperformed rats at long SOAs (greater than 100 ms). Moreover, rats produced faster responses compared to humans. The reaction time data further revealed key differences in decision process across the two species: at longer SOAs, reaction times increased in rats but decreased in humans. Finally, drift-diffusion modeling allowed us to isolate the contribution of various parameters including evidence accumulation rates, lapse and bias to the sensory decision. Consistent with the psychophysical findings, the model revealed higher temporal sensitivity and a higher lapse rate in rats compared to humans. These findings suggest that these species applied different strategies for making perceptual decisions in the context of a multimodal TOJ task.

## 1. Introduction

Rapid perceptual decision-making is often key to survival, for example a rat might need to quickly respond to the rustling of leaves to escape a lurking cat. To achieve rapid decisions, the brain needs to process the incoming sensory information, often requiring integration across multiple modalities, to generate an accurate percept and plan and execute the appropriate motor action, often all happening within several hundred milliseconds. The temporal precision of perceptual decisions vary across species; for instance electric fish can resolve 1 μs temporal disparity (Rose and Heiligenberg, 1985; Kawasaki et al., 1988) while echolocating bats can discriminate echo delay changes of 0.4 μs, corresponding to 0.07 mm distance (Moss and Schnitzler, 1989). The resolution at which temporal information is perceived by different species is key to survival and relates to a number of factors including body size and metabolic rate (Healy et al., 2013). For example, fast visual processing with high temporal acuity is necessary for small passerine birds which are high speed flyers that feed from fast moving prey (Boström et al., 2016).

We know that intersensory interactions occur when information from different senses arrives temporally or spatially coincident or near coincident in the brain (Zampini et al., 2005a; Keetels and Vroomen, 2012). The brain must faithfully represent the spatial and temporal features of the external world in patterns of neuronal activity for reliable perception. The temporal features of stimuli such as duration, interval and order of arrival carry crucial information for many aspects of perception (Buonomano and Maass, 2009). For instance, speech (Shannon et al., 1995) and reading (Farmer and Klein, 1995; Hairston et al., 2005; Vandermosten et al., 2010; Wallace and Stevenson, 2014) are disrupted if the temporal information is perturbed. These abnormalities are mainly generated by an extended temporal binding window (Wallace and Stevenson, 2014), for example, in autism (Boer et al., 2013; Stevenson et al., 2014) and schizophrenia (Haß et al., 2017; Stevenson et al., 2017). In the same way, temporal information is critical for how non-human animals, such as frogs, birds and monkeys, can use vocalization for communication (Mauk and Buonomano, 2004).

Discriminating the order of stimuli is a key aspect of temporal processing, which is commonly measured using the well-known temporal order judgment (TOJ) paradigm. In this paradigm, the interval between the onset of two stimuli, also referred to as the stimulus onset asynchrony (SOA), is systematically varied, while requiring the participant to report which side the first of the two stimuli appeared (Boenke et al., 2009), or which modality was presented first (Spence et al., 2003; Zampini et al., 2003b). Using this procedure, one can identify the interval at which the subject can no longer reliably judge the temporal order of stimulus presentation, and this interval is referred to as the point of subjective simultaneity (PSS). TOJ is used to study the temporal processing of both unimodal and multimodal stimuli (Spence et al., 2003; Zampini et al., 2003b; van Eijk et al., 2008; Boenke et al., 2009). Performance depends on the temporal precision of sensory processing within each modality (Keetels and Vroomen, 2012). Much is known about temporal order judgment of multimodal stimuli in humans. For example several factors affect human TOJs including low level factors such as the intensity and duration of stimuli (Boenke et al., 2009), and their spatial disparity (Spence et al., 2003; Zampini et al., 2003b), as well as high level cognitive factors such as attention (Spence et al., 2001), adaptation (Fujisaki et al., 2004; Van der Burg et al., 2013) and the causal relationship between stimuli (Fornaciai and Di Luca, 2020). Another phenomenon which affects the temporal order discrimination, particularly in audiovisual stimuli, is known as multisensory integration (King, 2005; Mégevand et al., 2013; Bedard and Barnett-Cowan, 2016) which refers to integration of two modalities across time. As a result of integration, subjects fail to accurately discriminate the order of audiovisual stimuli or even report them as simultaneous. Audiovisual integration depends on the experimental paradigm (i.e., TOJ versus simultaneity judgment) in which the PSS is estimated (van Eijk et al., 2008; Stevenson and Wallace, 2013; Bedard and Barnett-Cowan, 2016). In order to be perceived as simultaneous, often the visual stimulus has to precede the auditory stimulus (Zampini et al., 2003a; van Eijk et al., 2008; Leone and McCourt, 2015), indicating an inherent bias in humans.

Previous studies have shown that both rats and humans accumulate evidence for perceptual decisions (Brunton et al., 2013; Hanks and Summerfield, 2017). In multisensory context, both species can combine information across auditory and visual modalities to increase accuracy of their choices (Raposo et al., 2012; Sheppard et al., 2013). Integration of sensory evidence across time is a common theme in mammalian decision making (Hyafil et al., 2022). At the behavioral level such integration is manifested in reaction times and accuracy of perceptual decisions in both rats and humans (Hecht et al., 2008; Hirokawa et al., 2011; Gleiss and Kayser, 2012; Hancock et al., 2013). However, these species may use different strategies in resolving perceptual decisions and represent different speed-accuracy trade-offs (Reinagel, 2013b; Shevinsky and Reinagel, 2019). Applying a drift-diffusion model of decision making in the context of visual motion discrimination, a recent study (Nguyen and Reinagel, 2022) has revealed that while variability in the starting point of diffusion process can explain findings in rats, variability in the drift rate can better explain the pattern of human data. In both humans and rats, a small proportion of incorrect choices occur even in easy trials, known as lapse. The rate of lapse seems to be independent of the strength of sensory evidence and mostly reflect lack of attention to stimuli (Wichmann and Hill, 2001; Su et al., 2020). However, a recent study suggested that lapse in rats better explain an exploratory behavior rather than delivering unattended responses (Pisupati et al., 2021). This brief review of evidence clearly reflects similarities and differences in ways that these species resolve perceptual decisions.

Fundamental similarities and differences are also observed in temporal processing in these species. For example, both rats and humans can detect temporal regularities in sound (Celma-Miralles and Toro, 2020). But, in a temporal bisection task humans bisect intervals near their arithmetic mean, whereas rats tend to do the bisection near the geometric mean (Church and Deluty, 1977; Wearden, 1991; Kopec and Brody, 2018). Furthermore, there are reports that humans represent time in a linear manner while rats’ timing behavior is more akin to a logarithmic representation of time (Yi, 2009) (also see Ren et al., 2020 for an opposite view). In delay discounting of reward, which depends on representation of time, the two species act in a similar way but humans seem to be able to wait for much longer durations (Vanderveldt et al., 2016). In addition to interval timing, rodents have also been investigated in order timing. In the first effort for introducing a rodent model of TOJ, Wada and colleagues trained mice to detect the temporal order of vibrotactile stimuli applied to the long whiskers with brief air puffs. They found that mice exhibited lower temporal resolutions compared to humans, and revealed a lateral bias (Wada et al., 2005, 2010). More recent studies in rats (Schormans et al., 2017; Schormans and Allman, 2018, 2019), however, showed that rats could reliably perform TOJ with audiovisual stimuli. The general pattern of findings was similar to humans, but a cross-species investigation was lacking in these studies.

Here, by using the TOJ paradigm in rats and humans, we compare the temporal precision with which these species can detect the order of presentation of simple stimuli across two modalities of vision and audition. To this end, we designed similar audiovisual TOJ paradigms in which rats and humans were required to report which stimulus modality was presented first. Our goal was firstly to better understand how multiple sensory inputs are labeled and reported in their relative temporal occurrence in humans and rats, and secondly to investigate and directly compare the limits of temporal integration across these species. Finally, we performed drift-diffusion modeling on the human and rat data to isolate the contribution of various parameters including evidence accumulation rates, lapse and bias to the sensory decision. This data allows us to better understand how the profile of multisensory processing and labeling generalizes across species. To date, non-invasive methods such as electroencephalography (EEG) and functional magnetic resonance imaging (fMRI) have provided insights about neural underpinnings of multisensory temporal processing in humans (Bushara et al., 2001; Bernasconi et al., 2011; Binder, 2015; Basharat et al., 2018). However, having a comparable rodent model performing in analogous behavioral paradigms is important. This model will provide opportunities for invasive electrophysiological and lesion studies to gain insight to the neuronal computations underlying temporal discrimination or integration of multimodal stimuli.

## 2. Materials and methods

### 2.1. Subjects

Eight male Wistar rats, ranging in weight from 180 to 200 g were used in this study. Rats were 6-8 weeks old at the beginning of the training and 5-6 months old at the time of testing. They were housed in a well ventilated room on a 12-h dark/light cycle where lights were turned off at 7:00 P.M. Rats had *ad libitum* access to food and free access to water for 2 h each day after each training or testing session. Their weight was monitored throughout training to ensure that they continued to gain weight at a normal rate. In human study, ten participants (six females) aged 26-36 years (mean age of 31.6 years) were recruited to take part in this experiment. All participants were right-handed with normal or corrected to normal visual acuity and reported normal hearing. One participant was one of the authors of this paper, two of them had experience in psychophysical experiments and the others were naive to such experiments and the purpose of this study. All gave written informed consent prior to taking part in this study. Human experiment was conducted in line with the *Declaration of Helsinki* (Holm, 2013) and animal experiment was conducted according to *Guide for the Care and Use of Laboratory Animals* (Albus, 2012). All experiments were approved by the Ethics Committee of Kerman University of Medical Sciences (IR.KMU.REC.1401.151). For reporting the details of animal experiment we used ARRIVE guidelines (Percie du Sert et al., 2020).

### 2.2. Apparatus and procedure in rat experiment

#### 2.2.1. Apparatus

Rats were trained and tested in a custom-made wooden operant chamber, measuring 35 cm (length) × 25 cm (width) × 35 cm (height) which was placed in a dark and sound-attenuating box. The chamber had a central hole (1.5 cm depth) in the middle of its front wall, 4 cm above the floor to serve as a nose-poke area. The rats received liquid rewards (sucrose 5%) following correct responses in both training and testing sessions from two copper spouts, positioned on either side 5 cm away from the nose poke area. The spouts and nose poke used a capacitive sensor for detecting lick contacts. The capacitive sensors were connected to an arduino interface and were read by a MATLAB program at a high temporal resolution (>1 sample per ms). Animals’ contact with the spout or the metal piece at the end of the nose-poke hole, resulted in a sharp increase in the capacitance of the circuit. Applying a simple threshold allowed precise detection of behavior (nose-poke or lick at the spout). The auditory stimulus (4,500 Hz, 72dB at 5 cm distance) was presented from a speaker (8Ω, 0.5 W) 5 cm (center to center) above the nose-poke hole while the visual stimulus (3,200 cd/*m*^2^at 5 cm distance) was presented from a 5 mm white transparent LED located in the center of the front wall, 6 cm (center to center) above the nose-poke hole. Both stimuli had the same duration (20 ms). All procedures including stimulus presentation, measurement of behavioral responses and reward delivery were controlled by a MATLAB program and through an Arduino interface board. During the training and testing sessions the chamber was lit using a 9 W red LED bulb for video recording.

#### 2.2.2. Procedure

**FIGURE 1:**
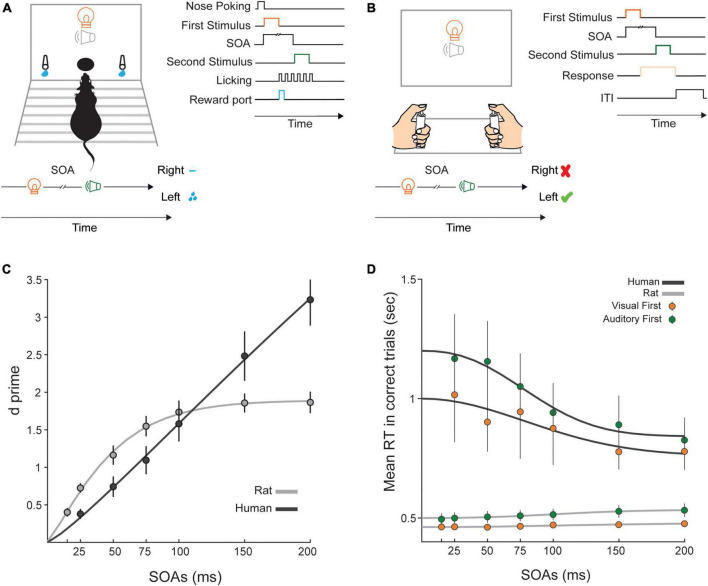
Temporal discriminability and reaction time in the audio-visual TOJ paradigm. **(A)** Schematic illustration of the experimental setup in rats. (Left) In the middle of the front panel of the testing chamber, there was a central hole for nose pokes. One LED and one speaker were positioned above the nose poke area. On either side of the nose poke area there were two spouts for collecting rewards (0.02 ml sucrose 5%). Also, schematic representation of a visual-leading trial in a subgroup of rats indicates that visual choices are mapped to the left spout. For another subgroup, a reversed contingency (not shown) was applied. (Right) Each trial included an auditory and a visual stimulus which were presented at a specific temporal delay (SOA) from each other. Each trial started with a nose poke that triggered the first stimulus presentation followed by the presentation of the second stimulus after the specific SOA. Rats could respond after the presentation of the first or the second stimulus by moving towards the left or right spout. **(B)** Schematic illustration of the human setup. (Left) Participants sat in front of a panel which had a central speaker and an LED. Left and right response keys, 45 cm apart, recorded participants’ responses. (Right) Each trial included one auditory and one visual stimulus which were presented at a specific SOA. Participants could respond after the presentation of the first or the second stimulus by pressing the right or left keys. **(C)** *d’* as a function of SOA. Higher values indicate more accurate detection of the temporal order of stimuli. Black circles indicate the averaged *d’* value for humans and the black line shows the fitted curve to *d’* values across SOAs. Gray circles indicate the averaged *d’* value across rats and the gray line shows the fitted curve to *d’* values across SOAs. **(D)** Average reaction time (RT) of correct responses across rats and humans. Green color indicates RT of auditory first trials and orange color shows RT of visual first trials. Black lines show fits to human data and gray lines show fits to rat data. Error bars indicate ± SEM across subjects.

In these experiments, we aimed to quantify the temporal precision with which rats and humans could perform TOJ of bimodal (audio-visual and visuo-auditory) stimuli. To this end, both stimuli were presented from a central point in front of the subjects (Figure 1A). We determined differences between humans and rats by comparing psychophysical measures between the two species. The task was designed in a similar manner across the two species and required subjects to choose the correct side, based on the modality of the first stimulus; for half of the subjects the left side was assigned as the correct side for auditory-first trials and the right side was assigned as the correct side for the visual-first trials. For rats, the final test data was collected in 8-12 sessions. The sessions were relatively short (maximum of 30 min) and rats initiated trials at their own pace. An average of 257 ± 34 trials were included in data for each SOA.

### 2.3. Training of rats

#### 2.3.1. Shaping to obtain the reward

Rats were initially habituated to the chamber, and learned to receive the reward from both spouts. Assignment of the modalities to the left/right spouts started at this stage such that responses (licks) at each spout were accompanied by presentation of a stimulus in predetermined modality. For example, a rat would receive an auditory tone each time it licked at the right side and would see a visual flash each time it licked at the left spout.

#### 2.3.2. Modality discrimination of unimodal stimuli

In this stage rats were trained to learn the assignment of modalities to left and right sides. For each modality, the left/right assignment was counterbalanced across rats. Rats initiated each trial by a nose poke into the central aperture (Figure 1A right), which led to the presentation of either auditory or visual stimulus for 20 ms. The stimulus was then followed by presentation of a cue (response cue) above the correct reward spout, e.g., a right cue for the visual stimulus and a left cue for the auditory stimulus. For each modality we used the same stimulus as the response cue; either a flickering visual stimulus (3 s/cycle) or repeated beeps (3 s/cycle). Visual and auditory response cues were presented from an LED and a speaker, the same as those used for presentation of test stimuli. Reward was only available on the cued side and was given even if this was not the animal’s first choice. The response cue remained until the reward was collected. No penalty was delivered upon incorrect first choices initially; however, once the criterion performance (>75% correct first choice for two consecutive days) was achieved, incorrect first choices led to termination of the trial without reward. After achieving the criterion performance in the new condition, the central stimulus was no longer accompanied by presentation of the response cue, and only correct first choices were rewarded.

#### 2.3.3. Modality and order discrimination of bimodal stimuli

At this stage, we initially presented bimodal stimuli with long SOAs (e.g., 900 ms) and then progressively reduced the SOA to 15 ms across training. Here, only correct first choices were rewarded. Incorrect first choices resulted in the termination of the trial without any reward. No other punishment was applied. In initial stages, reduction of SOA was performed through 50 ms steps, down to 100 ms SOA. In the final stages, SOA was reduced from 100 to 75, 50, 25, and then 15 ms. Across the initial stages, passing through steps was dependent on reaching the criterion performance (>75% correct first choice for two consecutive days) in individual rats. However, for SOAs lower than 100 ms (i.e., 75, 50, 25, and 15 ms), there was no criterion applied. In each session only the most recent SOA was applied. Following this stage, rats were required to choose the reward spout, corresponding to the side of the modality of the first stimulus. A 3-s inter-trial interval (ITI) was applied between trials. The ITI was measured after the rat responded to the previous trial. Supplementary Figure 1A illustrates how accuracy (averaged across 8 rats) systematically decreased as the SOA decreased across consecutive training stages. After learning the TOJ task with individual SOAs, we introduced intermixed SOAs within a single session. To habituate animals to this phase, we started with three long SOAs (200, 150 and 100 ms) presented in a pseudorandom order. After achieving the criterion performance, shorter SOAs were added step by step until rats could perform the task with all SOAs randomly interleaved in a single session. After stable performances (70 ± 3% accuracy in 150 and 200 ms SOAs across 2-3 sessions) rats were ready for the testing. Overall, the training procedure took 90-120 days.

### 2.4. Apparatus and procedure in human experiment

For testing humans in this study, participants sat in front of an experimental panel similar to that used for rats. The distance between the experimental panel and the participant’s eyes was ∼80 cm. For collecting responses (thumb presses in Figure 1B), two response keys were positioned 45 cm apart on the left and right sides. Each trial included a visual and an auditory stimulus, the same as those used in the rat experiment, that were presented centrally from the panel at the level of participants’ eyes. The participants initiated the first trial by pressing either the left or right key. The remaining trials started following delivering responses in the previous trial. Right/left keys were assigned for auditory-first/visual-first trials. Following response to a trial, a 3 s ITI was applied. Before commencing the test, participants were introduced to the setup, the position of the fixation point, the presentation of stimuli and how to use keys for responding. In the next step, 120-240 trials were presented for familiarization and training in a silent dark room which were excluded from analysis. The main data collection started after subjects indicated they were fully familiar with the paradigm and ready to do the task. The data was collected across 5-6 blocks of trials, in two days (2-3 blocks per day) and a 10-15 min break was introduced between blocks of each day. If a participant felt tired (or bored) during a block, we reduced the number of trials within that and subsequent blocks. This meant that different participants completed a different number of trials in total. Our criteria were to obtain a minimum of 85 trials per SOA from each participant. An average of 92 ± 6 trials was collected for each SOA.

The asynchrony between the onset of bimodal stimuli varied from 15 to 200 ms in rats and from 25 to 200 ms in humans. The minimum SOA that rats could distinguish better than chance was 15 ms with an average performance of 58 ± 3% (mean ± standard deviation) across rats (Supplementary Figure 1B). In our pilot testing, human participants reported difficulty in determining the temporal order of stimuli at the 25 ms SOA. We therefore did not include the 15 ms SOA for the human participants. The initial observation was confirmed once we ran the full experiment: unlike rats that could all perform SOA of 25 ms better than chance, only 5 of 10 human participants performed better than chance at the 25 ms SOA (binomial test, *p* < 0.05).

### 2.5. Data analysis

A common analytic approach was used for rat and human experiments. In both experiments, subjects reported the modality of the first stimulus. To quantify the behavioral performance at various SOAs, we applied the Receiver Operating Characteristics (ROC) analysis from Signal Detection Theory (Green and Swets, 1966). Accuracy was calculated relative to visual modality. Accordingly, the ‘hit rate’ was defined as the proportion of visual choices in visual-first trials. The “miss rate” was the proportion of auditory choices in visual-first trials. The “false alarm rate” was defined as the proportion of visual choices in auditory-first trials. And finally, the “correct rejection rate” was the proportion of auditory choices in auditory-first trials. We also used hit and false alarm rates to quantify discriminability (*d’*) at each SOA using the following equation in each subject:


(1) d⁢′=Z⁢(H)-Z⁢(F⁢A)

where, H is the hit rate, FA is the false alarm rate, and Z is the inverse of the cumulative Gaussian distribution function. Higher *d’* values indicate better temporal discriminability. The hit and false alarm rates for individual SOAs are depicted in the Supplementary Figure 2. In Supplementary Figure 1B, we also quantified accuracy in each SOA by averaging across the hit and correct rejection rates. An exponential function (Equation 2) was fitted to the data point of *d’*, accuracy and reaction time.


(2) $$0.5+\left(1-0.5-a\right)\times \left(1-e^{{\left(-\frac{x}{b}\right)}^{c}}\right)$$

For analysis of reaction time, we only included correct trials in which responses were generated sooner than 4 sec after stimulus presentation. The values beyond this limit were considered as potentially not a direct response to that trial and were therefore excluded from analysis for both species. The number of excluded trials was relatively small for most humans and rats (the median number of excluded trials was 3 for rats and 5 for human participants). We excluded incorrect trials in calculation of reaction time because these trials are unlikely to represent a correct reaction to the stimulus. In each condition for each rat, (i.e., 6 SOAs and 2 modalities), we pooled trials from all testing sessions and calculated the median of the distribution as reaction time for that condition. We have also represented reaction time without exclusion of long outliers, and for both correct and incorrect trials in Supplementary Figure 3.

A sigmoidal function (Equation 3) was fitted to responses at various SOAs using nonlinear least squares regression for all subjects to characterize the psychometric function (Wichmann and Hill, 2001; Keetels and Vroomen, 2012). Parameters of λ and γ are lower and upper asymptotes of psychometric function which are lower and upper lapse rates, respectively. Generally, such gaps show stimulus-independent or unattended erroneous responses when discrimination is easy. σ parametrizes the slope of the sigmoidal curve which shows sensitivity to temporal asynchronies. A higher slope indicates higher sensitivity in temporal order judgment. Finally, μ describes *x* value at the midpoint of psychometric function which indicates point of subjective simultaneity (PSS). The PSS is the temporal difference between two stimuli at which the subject is equally likely to report either modality as being first. In addition to slope, as an index of sensitivity we also estimated just noticeable difference (JND), where possible. The estimated value (mean ± SEM) was 0.067 ± 0.02 and 0.081 ± 0.01 in rats (*n* = 5) and humans (*n* = 10), respectively. Note that JND can reveal sensitivity in a broad range of SOAs (25 and 75% visual choices) whereas the slope can be more informative at very short SOAs.


(3) $$\lambda +\left(1-\lambda -\gamma \right)\times 0.5\,e\,r\,f\,\left(\frac{-\sigma \,\left(x-\mu \right)}{\sqrt{2}}\right)$$

To incorporate decision accuracy and reaction time into a single framework, we used drift diffusion modeling (DDM). Drift diffusion represents a class of integrator models, in which evidence in favor of each choice is accumulated until one of two decision thresholds (boundaries) is reached and a decision is made. The DDM was implemented using PyDDM simulator^1^. All SOAs (both auditory and visual first) were used in the same fitting, consistent with the most common approach in the literature (Palmer et al., 2005; Shinn et al., 2020).

Our approach for statistical analysis was to find out which factors affected performance in discrimination of temporal order of audiovisual stimuli and to examine whether these factors differ across the two species. To this end, we applied a mixed repeated measures ANOVA to either *d’* and reaction time in order to examine the effect of SOA and to elucidate species differences. For reporting statistical details where the sphericity (Mauchly test) was violated, degrees of freedom were corrected according to Greenhouse-Geisser Correction in ANOVAs. For statistical comparison of psychometric parameters (i.e., slope of psychometric function, PSS and lapse rate) and parameters derived from drift-diffusion modeling between two species, we used an independent-samples *t*-test in either case. In independent-samples *t*-test we checked homogeneity of variances using Levene’s test and in cases that homogeneity was violated we accordingly adjusted statistical details. For all statistical analysis, we used an IBM SPSS statistical package. The *p* value < 0.05 was considered as significant. We wrote code in MATLAB (R2021b) for extracting data and analysis including the sigmoidal fits and estimation of the fit parameters.

## 3. Results

We implemented a similar TOJ task in rats and humans to test the temporal precision with which these species detect the order of presentation of simple stimuli across two modalities of vision and audition (Figures 1A, B). Each trial contained a visual and an auditory stimulus, and subjects reported which modality was presented first. Here we begin by presentation of results with regard to the temporal discriminability derived from analysis of signal detection theory (Green and Swets, 1966), and then proceed with analysis of reaction time and parameters derived from psychometric function. Finally, we present parameters derived from fitting the drift-diffusion model to the data.

### 3.1. Temporal discriminability

Here we aimed to determine the similarity and differences of performance across the two species by measuring temporal discriminability (*d’*) in both species for all SOAs. We used hit (H) and false alarm (FA) rates (see Data analysis for details) in each subject to quantify *d’* at each SOA using the equation *d’* = Z (H) – Z (FA), where Z is the inverse of the cumulative Gaussian distribution function. Higher *d’* values indicate better temporal discriminability. Figure 1C shows that in rats, *d’* gradually increased from 25 to 100 ms SOA and reached a plateau in SOAs longer than 100 ms. In humans, on the other hand, the increase in *d’* by increasing SOA revealed a more linear trend. We used a mixed-measures ANOVA (SOA, within-subject factor × Species, between-subject factor) to examine the effect of SOA on *d’* value and to examine any statistical differences between humans and rats. The effect of Species was not significant (*F* (1, 16) = 0.17; *p* = 0.68, *ηp^2^* = 0.01). This indicates that, when collapsed across the SOAs, the average *d’* and therefore, the overall temporal discriminability was comparable between humans and rats. However, the main effect of SOA was highly significant (*F* (1.98, 31.66) = 78.62; *p* = 0.001, *ηp^2^* = 0.83), showing that the interval between stimuli determined their temporal discriminability. Post-hoc analysis with Bonferroni correction revealed significant differences between all pairs of SOA. The highest p value was 0.009 indicating difference between 50 and 75 ms SOAs. Importantly, there was a significant interaction between the SOA and Species factors (*F* (1.98, 31.66) = 19.13; *p* = 0.001, *ηp^2^* = 0.54) indicating that the relationship between discriminability and SOA does not follow the same pattern in two species. Rats exhibited higher *d’* in short SOAs (25 ms, 50 ms, and 75 ms) whereas humans outperform rats at higher SOAs (>100 ms) (Figure 1C). To quantify this significant interaction, we conducted *post-hoc* independent-samples *t*-test separately for the lowest and highest SOAs. At 25 ms SOA, the *d’* was significantly higher in rats (*t* (16) = 3.69, *p* = 0.004). On the other hand, at 200 ms SOA, *d’* was significantly higher in humans (*t* (11.93) = −3.67, *p* = 0.003).

To further investigate the effect of modality on performance, we included accuracy of choices in visual-leading (i.e., hit (H) rate) and auditory-leading (i.e., correct rejection (CR)) trials in a mixed-measures ANOVA using SOA (6 SOAs) and Modality (H: visual/CR: auditory) as within-subject factors and Species as a between-subject factor. The main effect of Modality (*F* (1, 16) = 4.08; *p* = 0.06, *ηp^2^* = 0.20), its interaction with Species (*F*(1, 16) = 0.07; *p* = 0.79, *ηp^2^* = 0.005) and SOA (*F*(1.4, 22.44) = 0.39; *p* = 0.6, *ηp^2^* = 0.024) were not significant. These findings indicate that in both species, order of modalities did not affect discrimination accuracy across the range of SOAs.

### 3.2. Reaction time

We next asked whether reaction times were also systematically different between the two species. In a decision process, a trade-off often exists between speed and accuracy, whereby difficult decisions often correspond to longer reaction times (Chittka et al., 2009). We calculated reaction times, from onset of first stimulus to the moment of response, to examine how speed of choices was affected by SOA and modality. In this study, we asked human participants to focus on accuracy rather than speed. Similarly, we did not introduce a limited response window for rats and therefore did not encourage fast responses across training and testing sessions. Figure 1D shows reaction time in each species for two conditions: visual-first and auditory-first trials. To test differences in reaction time across species, we applied SOA (6 SOAs) and Modality (visual/auditory) as within-subject factors and Species as a between-subject factor in a mixed-measures ANOVA. The effect of Species was significant (*F* (1, 16) = 9.28; *p* = 0.01, *ηp^2^* = 0.37). However, given the differences in the apparatus and the type of response required from the two species, the absolute differences in reaction time are hard to interpret. The main effect of Modality was significant (*F* (1, 16) = 8.3; *p* = 0.01, *ηp^2^* = 0.34): we observed shorter reaction times in visual-first trials. Interaction between the effects of Modality and Species was not statistically significant (*F* (1, 16) = 1.67; *p* = 0.21, *ηp^2^* = 0.09) (Figure 1D). The main effect of SOA was significant (*F* (1.43, 22.85) = 4.15; *p* = 0.04, *ηp^2^* = 0.21), which means that the temporal interval between stimuli had an effect on reaction time. Importantly, there was a significant interaction between the SOA and Species factors (*F* (1.43, 22.85) = 5.73; *p* = 0.02, *ηp^2^* = 0.26) indicating that SOA affected reaction times differently in the two species. Interaction between SOA, Species and Modality (*F* (1.81, 29.02) = 0.8; *p* = 0.45, *ηp^2^* = 0.05) was not significant. Also, no significant interaction was revealed between the effects of Modality and SOA factors (*F* (1.81, 29.02) = 0.66; *p* = 0.51, *ηp^2^* = 0.04). Visual inspection of data (Figure 1D) revealed that reaction time increased by increasing SOA in rats, whereas a reverse pattern exists in humans. To examine whether a significant trend exists in reaction time as a function of SOA in each of visual-first and auditory-first choices, we applied a linear regression analysis using reaction time as dependent variable and SOA as independent variable. In rats, both the visual-first (Pearson’s *r* = 0.97; *p* = 0.001) and auditory-first (Pearson’s *r* = 0.97; *p* = 0.001) choices were positively correlated with SOA. In humans, however, a significant but negative correlation was found in both the visual-first (Pearson’s *r* = −0.92; *p* = 0.009) and auditory-first (Pearson’s r = −0.96; *p* = 0.002) choices. Overall, these findings indicate that while for humans, reaction times increased with task difficulty, the opposite relation was observed in rats.

### 3.3. Psychometric parameters

**FIGURE 2:**
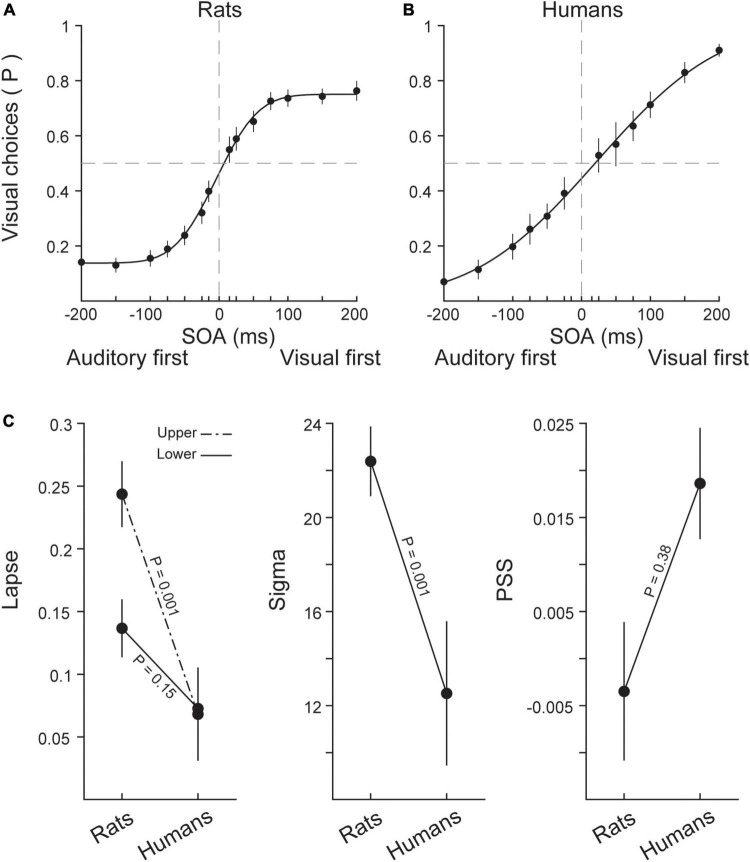
Psychophysical quantification of performances in rats and humans. **(A)** The averaged responses to bimodal stimuli in different SOAs in rats. Positive SOAs represent trials in which the visual stimulus was presented first. Negative SOAs represent trials in which the auditory stimulus was presented first. Black circles indicate the average responses across eight rats, and the black line shows the sigmoidal fit (cumulative Gaussian function) to performance values. The mean of the sigmoidal fit represents the point of subjective simultaneity (PSS) and the sigma represents the temporal sensitivity (slope of the sigmoidal function). **(B)** The average performance across human subjects (*n* = 10) and the fitted sigmoid. Color conventions are the same as **(A)**. **(C)** The average values of upper (visual) and lower (auditory) lapse rates, slope (temporal sensitivity) and PSS plotted for rats and humans. Error bars indicate ± SEM across subjects.

In this section, for obtaining performance measures and examining the similarities and differences between the two species, we applied psychometric function to responses of individual rats and humans. To this end, we fit a cumulative Gaussian sigmoid curve to accuracy rates for rats and humans. These methods allowed us to isolate key parameters of the performance including (i) the temporal sensitivity which is captured by the slope of the sigmoidal function (ii) the interval at which the two stimuli are perceived as simultaneous (PSS) and (iii) the lapse rate which represents the probability of delivering unattended responses, regardless of the difficulty of stimulus. Figures 2A, B shows psychometric function fitted to the averaged data of rats and humans, respectively. Figure 2C illustrates the three parameters of interest for each species. Rats exhibited a larger upper lapse rate (independent samples *t*-test: *t* (16) = 4.75, *p* = 0.001) which indicates that rats delivered more unattended or random responses specially in visual first trials compared to humans even when detecting temporal order of stimuli was easy. However, the lower lapse rate was similar in two species (independent samples *t*-test: *t* (16) = 1.52, *p* = 0.15). There was a significant difference in slope of the psychometric function fitted to human and rat data (independent samples *t*-test: *t* (16) = 4.64, *p* = 0.001) indicating that slope of psychometric function was different in two species. This revealed temporal sensitivity of rats was higher than humans. Therefore, rats compared to humans are able to distinguish stimuli with smaller temporal intervals from each other. In other words, rats had higher sensitivity to changes in SOAs. No significant difference existed between PSS of humans and rats, determined by independent samples *t*-test (*t* (11.04) = −0.908, *p* = 0.38). In rats, the average PSS (−4 ms) was not different from zero (one-sided *t*-test: t (7) = 0, *p* = 0.5), showing that they were unbiased to auditory and visual stimuli. Finally, in humans, the average PSS was +19 ms which was not statistically different from zero (one-sided *t*-test: t (9) = 0, *p* = 0.5), revealing no evidence for a modality bias in humans.

### 3.4. Drift diffusion model

**FIGURE 3:**
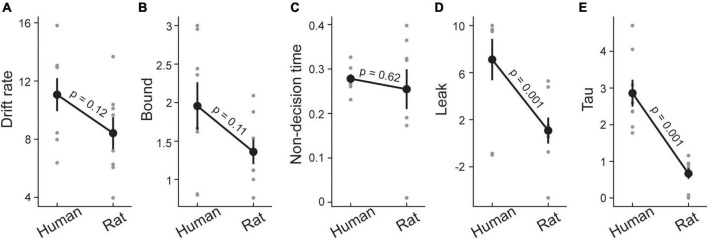
Drift diffusion model in rats and humans. Five parameters of drift diffusion including drift rate **(A)**, decision threshold or bound **(B)**, non-decision time **(C)**, leak **(D)**, and collapsing bounds or tau **(E)** illustrated for rats and humans. Gray circles show each parameter for individual subjects and black circles depict the average of each parameter across subjects. Error bars indicate ± SEM across subjects.

So far we have found that rats have higher performances at short SOAs compared with humans, but exhibited lower performances at longer SOAs. There were no systematic differences between the two species in the point of subjective simultaneity. One difference that stood out in the psychometric function was the high lapse rate of rats compared to humans. The more impulsive behavior in rats, that may be partially due to task demands, could explain their high lapse rate. This is also consistent with the reaction time data where rats showed systematically faster responses and did not decrease their speeds with increasing task difficulty. To provide a more quantitative understanding of the underlying mechanisms that determined these sensory decisions in rats and humans, and to incorporate decision accuracy and reaction time into a single framework, we used drift diffusion modeling (Palmer et al., 2005). Drift diffusion modeling (DDM) represents a class of integrator models, in which evidence in favor of each choice is accumulated until one of two decision thresholds (boundaries) is reached and a decision is made (Uchida et al., 2006; Ratcliff et al., 2016). One of the principal benefits of this approach is that it uses both accuracy and reaction time to fit the model, which should account for the speed/accuracy trade-offs suggested by the data so far. The DDM analysis therefore focused on trials where a behavioral choice was made in a time window that tightly followed the arrival of the sensory information (i.e., from 0.1 to 1.65 s after the stimulus presentation). We fit a DDM with five parameters using PyDDM. The *drift rate* reflects the rate of sensory information accumulation. The *decision threshold* determines the amount of sensory evidence that needs to be accumulated before the choice is made. The *nondecision time* is the portion of reaction time which is not related to the decision process (i.e., the afferent delay, and the motor execution times). The *leak* reflects the amount of sensory information that is lost over time. Finally, we fit a *collapsing bounds parameter (tau)* to capture the rat’s impulsivity where less evidence is needed at longer reaction times. Figure 3 illustrates the summary of the decision parameters for the human and rat data. Two parameters stand out as showing large differences between the species: the collapsing bounds parameter (*t* (16) = 5.58, *p* < 0.001) and the leak rate (*t* (16) = 2.91, *p* = 0.001). Rats also exhibited a trend towards a lower decision bounds (thresholds) compared to human participants (*t* (16) = 1.71, *p* = 0.11). Altogether, having a faster collapse of bounds and rate for information leak along with lower bounds could explain the earlier observation that rats had significantly faster reaction times across all SOAs, whereas humans showed longer reaction times with short SOAs. As rats’ bounds collapse more quickly, they tended not to delay their response at short SOAs despite the reduced evidence. This potentially explains why despite having higher lapse rates, rats exhibited a superior performance at low SOAs. Unlike the classic global dot motion paradigms, where sensory evidence continuously flows in, here the information is provided in a single time at the onset of the trial. Therefore, any delays in the decision can potentially corrupt the evidence through leakage. It is, therefore, unsurprising short SOAs where reaction times were longer corresponded to inferior performance in humans.

## 4. Discussion

We characterized the temporal precision with which rats and humans could detect the order of presentation of visual and auditory stimuli with a similar TOJ task in both species. We applied centrally-presented pairs of audiovisual stimuli to rats and humans at a range of interleaved SOAs. Both species exhibited high precision in distinguishing the temporal order of audiovisual stimuli with systematic similarities and differences in their parameters of performance. By applying principles of signal detection theory, we found that temporal discriminability (*d’*) systematically decreased by decreasing the SOA in both species. However, rats showed greater sensitivity at short SOAs (shorter than 100 ms) whereas humans showed greater performance at long SOAs (longer than 100 ms) (Figure 1C). We found that the rats’ higher lapse rate compared to humans could contribute to this profile of performance. A drift diffusion modeling further confirmed that rat’s superior performance at short SOAs was possibly due to faster collapsing decision bounds rather than higher drift rates. Such differences in performance profile may also reveal limitations of each species in detection of temporal mismatch between bimodal stimuli.

It is known that the resolution at which temporal information is perceived by different species depends on their body size and metabolic rate (Healy et al., 2013). For example, in a flicker fusion frequency task small passerines birds distinguished flickering stimuli at temporal precision of around 130 Hz, which is substantially higher than other vertebrates including humans (Boström et al., 2016). An organism’s sensory limitations impose critical constraints on how the organism can interact with its environment. In the current study, better temporal discriminability and accuracy in short SOAs in rats compared to humans support this assumption. On the other hand, lower accuracy in long SOAs in rats compared to humans might reflect the fact that humans primarily focused on accuracy rather than speed whereas rats maximized their reward gain not necessarily *via* increasing accuracy. For the current experiment, correct responses in rats were accompanied by a sucrose reward, therefore, incentive anticipation of reward probably may have promoted unattended impulsive responses (Donnelly et al., 2015; Marshall and Kirkpatrick, 2016) in a proportion of easy trials (i.e., long SOAs; greater than 100 ms).

In addition to accuracy, we also measured reaction time across SOAs and found that overall rats performed the task with shorter reaction times compared to humans, and this was despite the fact that rats needed to reach the lick ports rather than merely pressing a button. Shorter reaction time in rats may indicate that their strategy in performing the task is different from that of humans. It has been shown that in difficult choices, animals may prioritize responding more quickly when no punishment is expected (Chittka et al., 2009). For encouraging animals to reduce speed and increase accuracy one needs to manipulate task parameters including reward or punishments as reinforcers. For example bees sacrifice speed in favor of accuracy when quinine penalties are applied after error responses (Chittka et al., 2003). In our study, incorrect responses accompanied no reward but also no explicit punishment. It is therefore not surprising that rats focused on speed rather than accuracy in order to maximize reward gain. Another difference between the two species was in the dynamics of speed accuracy trade-off. Generally, the speed accuracy trade-off should be optimized given the specific demands of the environment. The speed accuracy trade-off has been studied for over a century in different species including house-hunting ants (Stroeymeyt et al., 2010), bees (Chittka et al., 2003), mice (Rinberg et al., 2006), rats (Kaneko et al., 2006; Reinagel, 2013a; Mendonça et al., 2020), and human (Herz et al., 2017) and non-human primates (Heitz and Schall, 2012; Hanks et al., 2014). In our study, rats performed the task with faster responses compared to humans. However, such fast responding was even more pronounced in shorter SOAs (i.e., difficult choices) whereas, in line with previous studies, in humans more difficult choices resulted in longer decision times (Figure 1D). For example, in a visual task involving detection of motion direction, increasing difficulty by decreasing fractions of coherently moving dots increases reaction time in monkeys (Roitman and Shadlen, 2002) and humans (Palmer et al., 2005). However, rats revealed a different strategy in performing motion discrimination (Shevinsky and Reinagel, 2019) and visual image discrimination (Reinagel, 2013a) tasks. Shevinsky and Reinagel (2019) designed an analogous motion discrimination task in rats and humans and in line with the current study they observed that while humans had longer reaction time at lower accuracy, rats exhibited shorter reaction time at lower accuracy (Shevinsky and Reinagel, 2019). On the other hand, in a tactile detection task involving whisker inputs, rats increase their sampling time in difficult trials to obtain more information about whisker deflection (McDonald et al., 2014). It is therefore not entirely clear whether the differences in speed accuracy trade-off reflect fundamental differences across species or are partially determined by sensory modality or task parameters.

Another difference between rodents and humans is the temporal window in which they accumulate evidence for making perceptual decisions. For example, rats need about 300 ms to decide between a mixture of two odors while only 35 ms more time is enough for making decisions in difficult trials (Uchida and Mainen, 2003). This time is reported as long as 80 ms in mice (Abraham et al., 2004). These results indicate rodents need a relatively small window of integration compared to what humans or monkeys need to accumulate evidence. In rats, the interplay between speed and accuracy also depends on the task. For example, in both olfactory identification (single pure odor) and olfactory categorization (mixture of two pure odor was presented) tasks, reaction time increased with task difficulty but with smaller trends in the categorization task (Mendonça et al., 2020). This indicates that categorization benefits less from temporal integration. Of great relevance to the context of the current study is TOJ of multisensory stimuli, which is rarely investigated in rats (Schormans et al., 2017; Schormans and Allman, 2018). As a common phenomenon in multisensory contexts, integration of modalities may improve target detection, but it interferes with tracking the order information (Miller and Schwarz, 2006; Mégevand et al., 2013). Indeed, integration of information across modalities hinder discrimination of their order in TOJ and therefore increases reaction time in difficult choices (i.e., short SOAs), although it is not a common theme across studies of this kind [e.g., see Leone and McCourt, 2015]. Also, a clear distinction exists between TOJ and simultaneity judgment tasks (Love et al., 2013; Bedard and Barnett-Cowan, 2016; Basharat et al., 2018). In contrast to TOJ, integration of modalities in simultaneity judgment, is in line with perceiving them as simultaneous, particularly in short SOAs, which come up with a reduction in reaction time (Matthews et al., 2016; Simon and Wallace, 2018). We found that in humans, reaction time increased with decreasing SOA, whereas an opposite pattern was observed in rats. Whether this discrepancy depends on the strategy that the two species used for discrimination of stimuli or the way that they deal with multisensory integration remain to be addressed in future studies. We also found shorter reaction times in correct visual-first choices in both species. This is not consistent with the observation that unisensory reaction time is faster in auditory than visual stimuli (Leone and McCourt, 2015; Bella and Silvestri, 2017; Harrar et al., 2017), and the fact that processing time of auditory modality is shorter than that of the visual modality. Whether a faster reaction in visual choices depends on the task demand and is specific to TOJ needs further investigation.

In this study, we applied a cumulative Gaussian function to data to quantify psychophysical aspects of human and rat performance, including bias or PSS, slope and lapse rate. PSS indicates the temporal delay at which the two stimuli are perceived as simultaneous: i.e., where the probabilities of visual-first and auditory-first choices are equal. We found a PSS of −4 ms in rats, but this was not significantly different from zero indicating that they were unbiased to visual and auditory stimuli. However, a recent study showed an average PSS of −8.8 ms across seven rats (Schormans et al., 2017). In humans the average PSS was +19 ms however, again this was not statistically different from zero. Despite the numerical difference, PSS was not significantly different between humans and rats. From previous studies we know that the value and sign (negative or positive) of PSS can vary with the intensity (Schormans and Allman, 2018), or duration (Boenke et al., 2009) of stimuli as well as with task conditions (Zampini et al., 2005b). Another key finding in psychometric parameters was the slope of the fitted sigmoid as a measure for temporal sensitivity. In our study, rats exhibited a higher slope and consequently higher temporal sensitivity at short SOAs compared to humans. Also, rats revealed a higher lapse rate compared to humans which predominantly was derived from a large visual lapse in rats (Figure 2C). In similar studies in rats (Schormans et al., 2017; Schormans and Allman, 2018, 2019), there is no report of lapse, so it remains to be clear in future studies whether this is an inherent bias in rats or depends on the experimental paradigm. The higher lapse rate along with shorter reaction time in rats indicates that two species have adopted different strategies in resolving the task. Consistent with our findings, a higher lapse rate has been reported in rats compared to humans in a motion discrimination task (Shevinsky and Reinagel, 2019) reflecting a species difference in speed-accuracy strategy so that rats’ reaction time strongly affects by elapsed time during a trial rather than strength of evidence (Reinagel, 2013b). When discussing drift diffusion model, we further elaborate the species differences in terms of decision strategy.

In addition to measuring accuracy and speed across the range of SOAs, we also applied a drift-diffusion model to investigate differences and similarities between decision process in humans and rats. As discussed earlier, making fast decisions is expected to come at the expense of reduced accuracy. A number of computational models including the stochastic accumulator models (Usher and McClelland, 2001; Smith and Ratcliff, 2004; Tavares et al., 2017; Roxin, 2019; Shinn et al., 2020) are used to quantify speed accuracy trade-off. These models allow us to isolate fundamental aspects of a decision making process, including the rate of sensory information, the arrival to a decision threshold for making choices, and the lapses and biases that influence sensory decisions. We applied the same model to our human and rat data to reveal fundamental similarities and differences by which these species make decisions regarding the temporal order of stimuli. To this aim, a variant of drift-diffusion model which considers collapsing boundaries for decision over time was fitted to the data. This model can capture the data in rats (Mendonça et al., 2020) and monkeys (Hawkins et al., 2015) when learning and training shape the decisions. Another reason for using this model was that we used a short range of asynchronies and therefore evidence with regard to stimuli’ order can be degraded over time, which corresponds to collapsing decision boundaries across time (Hanks et al., 2014; Voskuilen et al., 2016; Malhotra et al., 2018). This model assumes that in a given trial, the requirement of evidence for making decisions decreases as a function of time. Also, it has been shown that a cost exists for spending time on tasks in human and non-human primates because accumulation of information is a demanding process (Drugowitsch et al., 2012) and therefore collapsing boundaries prevent consuming limited cognitive resources. We found a similar picture, particularly in rats’ data, so that they revealed lower leakage, performed the task with shorter reaction times and outperformed humans in short SOAs. Unlike the aforementioned primate motion discrimination paradigm (Roitman and Shadlen, 2002; Hanks et al., 2014), where sensory evidence continuously flows in, here the information is provided in a single time at the onset of the trial. Therefore, any delays in the decision can potentially corrupt the evidence through leakage (Drugowitsch et al., 2012). Faster and more accurate choices in short SOAs in rats is also consistent with faster collapsing boundaries in rats. The model thus explained why despite having lower decision thresholds and higher lapse rates, rats maintained a higher level of performance compared to human participants, particularly in short SOAs.

To allow comparison between species, we designed an analogous TOJ task for humans and rats. However, important differences remain between the two paradigms and the training schedules, which should be taken into consideration when interpreting the findings. Humans could be instructed as to the requirements of the perceptual task, but rats needed an extensive training protocol to shape their behavior. The rats were rewarded for correct choices and therefore had a different level of motivation compared to human participants. The sucrose reward also produced an immediate feedback to the rats regarding the choice on every trial. We therefore cannot rule out the potential impact of feedback or difference in the duration of training/familiarization on the observed species differences in the current study. Additionally, the type of response required differed between the two species: rats responded by moving their body towards and licking from the reward spout, whereas humans performed a manual response. Another difference was the application of 15 ms SOA in rats but not in humans. The range of SOAs was determined based on performances. Human participants found temporal discrimination at the 25 ms SOA difficult. Indeed, only half of the human participants performed better than chance at this SOA, whereas all rats performed better than chance for the same SOA. We therefore extended the SOA for rats to also include the 15 ms interval which was the smallest that we could reliably implement. Finally, mapping auditory and visual modalities to the left/right side was equally counterbalanced across rats, whereas, counterbalancing was not the case in human subjects. All subjects (right-handed) in the visual-leading and auditory-leading trials responded to the left and right sides, respectively. However, we did not observe any systematic differences in reaction times, and lack of bias in the PSS implies that overall performance was not influenced by nonspecific factors such as handedness or any potential intrinsic left/right bias in subjects. Only a modality-dependent bias was observed for reaction time, such that the average reaction time was shorter in the visual choices. Because visual choices were mapped to the left hand, therefore shorter response times in the left/visual choices (compared to right-auditory choices) suggests the presence of a modality effect rather than a handedness bias which would have been expected to favor the right choices in right-handed individuals. Future studies could adjust the human and rat paradigms to remove some of the methodological differences here including the extent of training and the presence of feedback. These studies could also record signals in the form of EEG from humans and neural activity in behaving rats undertaking this task to investigate the neuronal mechanisms that underlie perceptual decisions in such multimodal contexts.

## 5. Conclusion

By implementing a similar psychophysical paradigm in rats and humans, we found that both species could reliably distinguish the temporal order of audio-visual stimuli based on the modality label. Rats showed superior performance at difficult trials, with SOAs shorter than 100 ms, whereas humans showed superior performance at easier trials, with SOAs greater than 100 ms. Rats exhibited a high temporal precision by performing better than chance even at the 15 ms SOA. Overall, rats produced faster responses compared to humans and did not increase their reaction time as difficulty increased. Despite their higher temporal precision, rats’ overall performance was hampered due to a high level of lapse rate and this was most evident at the easy trials with long SOAs. Despite a few similarities, our findings indicate that rats and humans apply different strategies in terms of speed and accuracy in resolving perceptual decisions when the difficulty of decisions varies across trials.

## Data availability statement

The raw data supporting the conclusions of this article will be made available by the authors, without undue reservation.

## Ethics statement

The studies involving human participants were reviewed and approved by the Ethics Committee of Kerman University of Medical Sciences. The patients/participants provided their written informed consent to participate in this study.

## Author contributions

FM, MA, and EA designed the initial project. FM performed the experiments. All authors contributed to the data analysis and interpretation. FM, SG, and EA designed and built the experimental apparatus and wrote code to control the stimuli and monitor the behavior. MT performed the initial modeling. FM and EA drafted the first version of the manuscript. All authors contributed to the subsequent revisions.

## Footnotes

1
https://github.com/murraylab/PyDDM
