# Supplementary material for: Temporal order judgment of multisensory stimuli in rat and human
Source: Front Behav Neurosci. 2023 Jan 12;16:1070452. doi: 10.3389/fnbeh.2022.1070452 (PMC9879721; doi:10.3389/fnbeh.2022.1070452)

**Supplementary Materials**


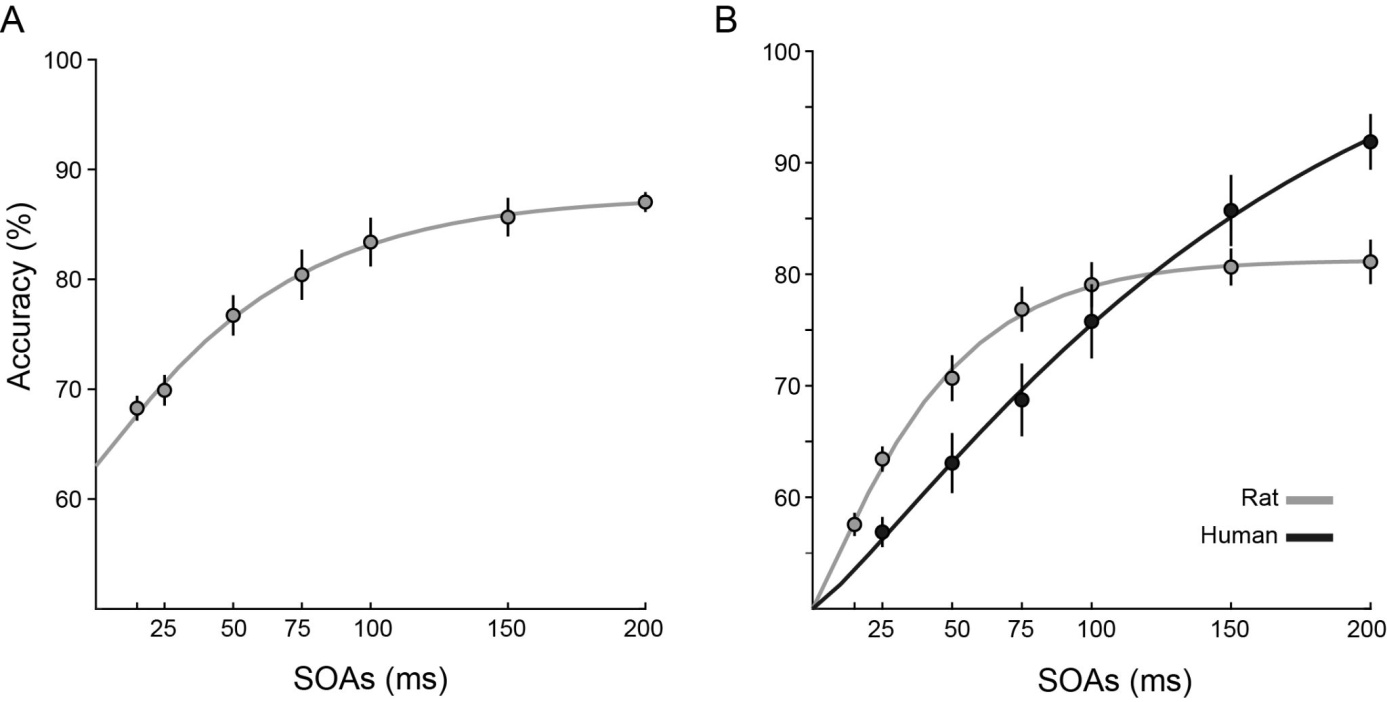


**Supplementary Figure 1. Accuracy of temporal order judgment.** (A) The average accuracy during the training phase of rats. In the training phase, rats experienced only a single temporal interval (SOA) in each session. Each circle indicates the average responses across eight rats in each SOA which is collected during decreasing SOA in consecutive sessions. Gray curve shows the fitted line to the data points. (B) The average accuracy during the testing phase of rats and humans. In the testing phase all SOAs were presented pseudorandomly in each session. Accuracy is quantified based on hit rates and correct rejections. Black circles indicate the averaged accuracy for humans (n=10) and black line is a fitted curve to the respective data points. Gray circles indicate the averaged accuracy across rats (n=8) and gray line is a fitted curve to the respective data points. Error bars indicate ± SEM across subjects.

*
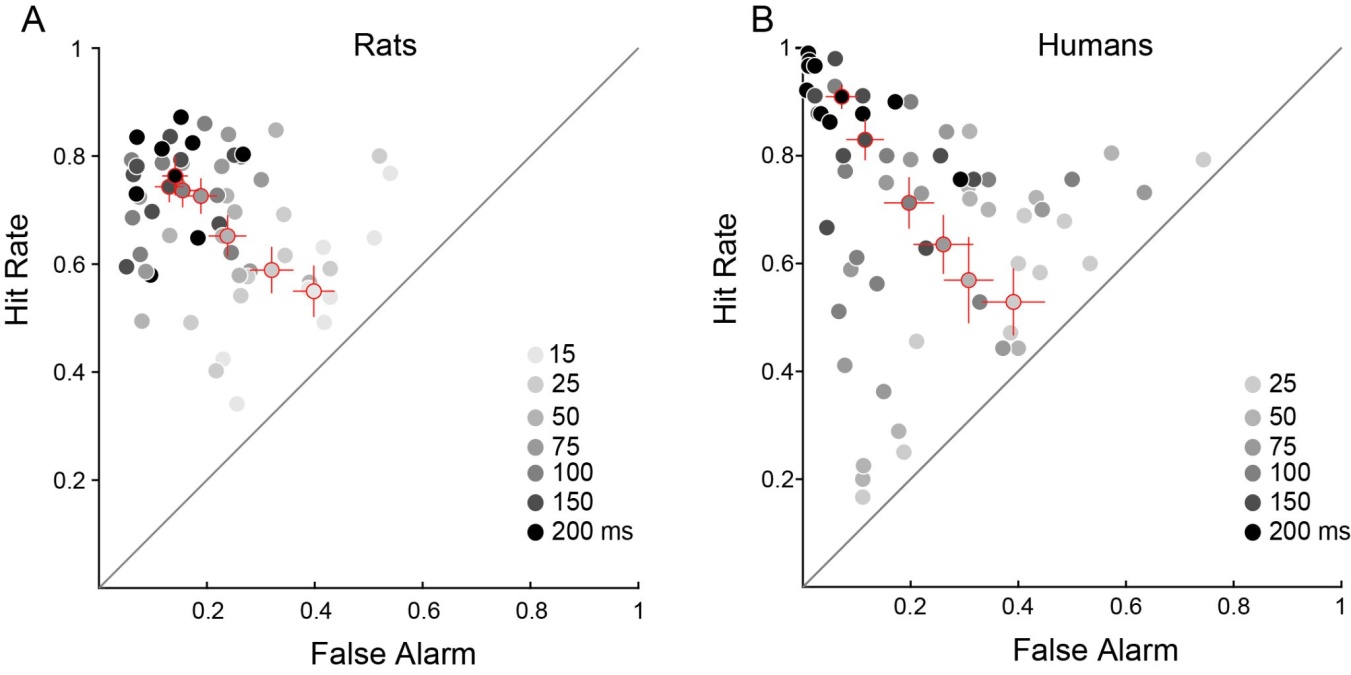
*

**Supplementary Figure 2. Receiver Operating Characteristics (ROC) analysis of rats and humans**. (A) False alarm and hit rate of individual rats in centrally- presented stimuli. Darker circles are related to longer SOAs. Red circles are the average hit rate and false alarm of subjects (n=8) for each SOA. (B) False alarm and hit rate of individual humans in centrally- presented stimuli. Darker circles are related to longer SOAs. Red circles are the average hit rate and false alarm of subjects (n=10) for each SOA. Error bars indicate ± SEM across subjects.

**
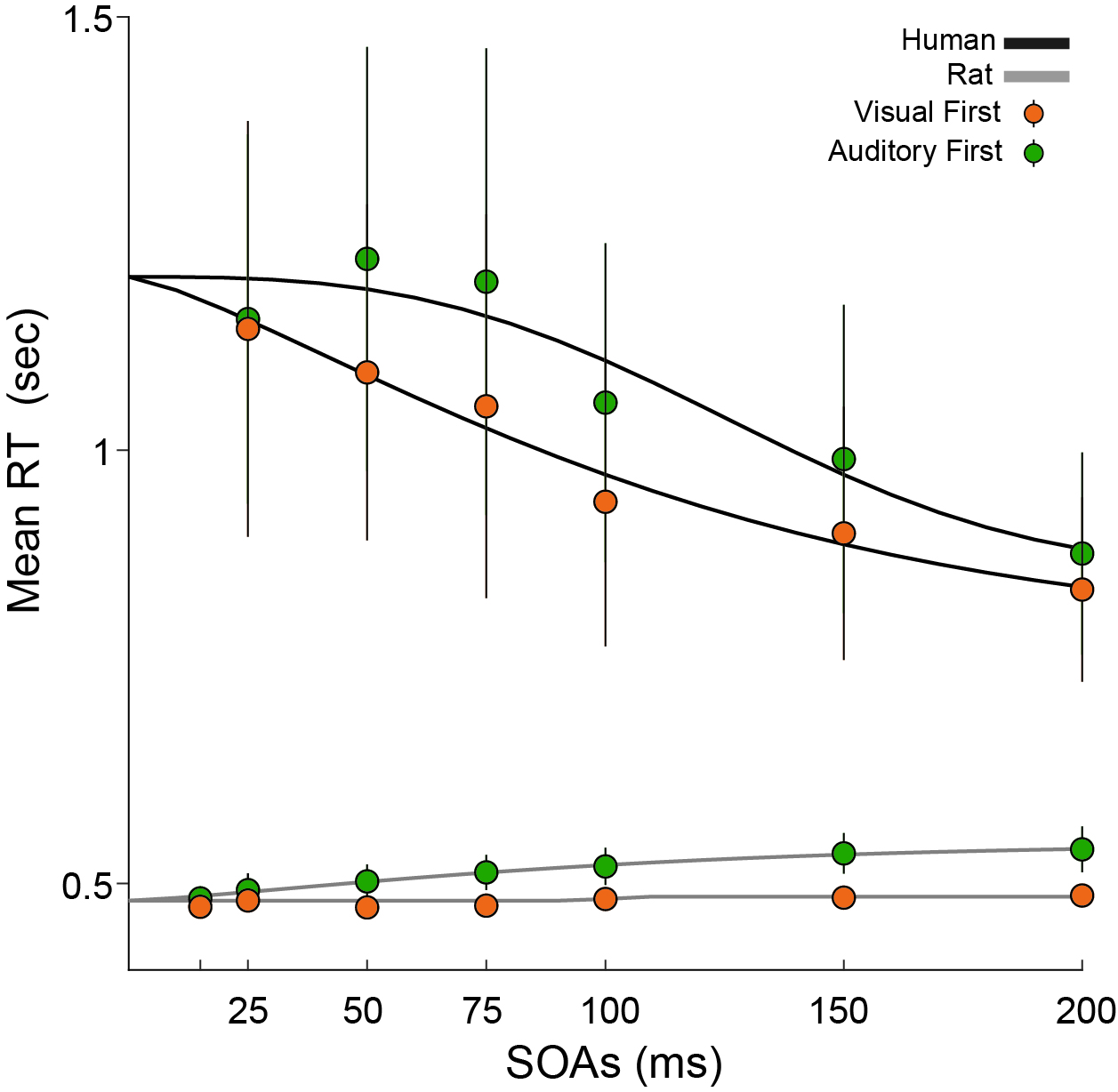
**

**Supplementary Figure 3. The average reaction time (RT) without exclusion of outliers and incorrect choices.**

**Supplementary Table 1. Accuracies in long SOAs across 3 sessions before commencing data collection in rats.**


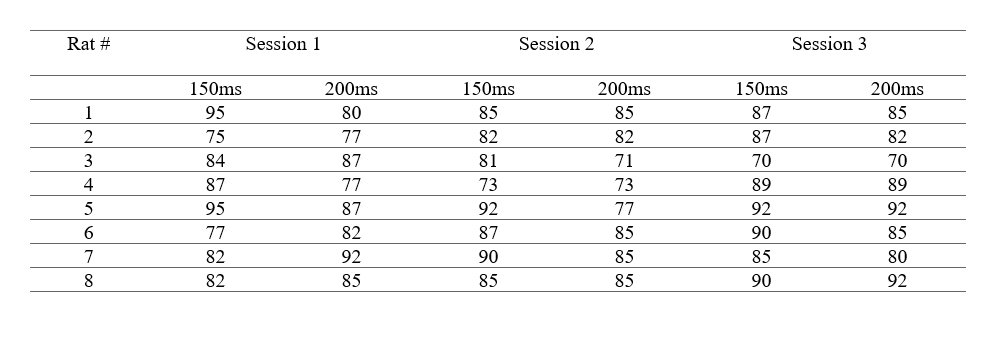

Supplement: Supplementary file 1 [file Data_Sheet_1.docx]
